# Sex-Related Differences in Long-Term Outcomes After Early-Onset Myocardial Infarction

**DOI:** 10.3389/fcvm.2022.863811

**Published:** 2022-07-04

**Authors:** Maddalena Ardissino, Adam J. Nelson, Giuseppe Maglietta, Guidantonio Malagoli Tagliazucchi, Caterina Disisto, Patrizia Celli, Maurizio Ferrario, Umberto Canosi, Carlo Cernetti, Francesco Negri, Piera Angelica Merlini, Marco Tubaro, Carlo Berzuini, Chiara Manzalini, Gianfranco Ignone, Carlo Campana, Luigi Moschini, Elisabetta Ponte, Roberto Pozzi, Raffaela Fetiveau, Silvia Buratti, Elvezia Maria Paraboschi, Rosanna Asselta, Andrea Botti, Domenico Tuttolomondo, Federico Barocelli, Serena Bricoli, Andrea Biagi, Rosario Bonura, Tiziano Moccetti, Antonio Crocamo, Giorgio Benatti, Giorgia Paoli, Emilia Solinas, Maria Francesca Notarangelo, Elisabetta Moscarella, Paolo Calabrò, Stefano Duga, Giulia Magnani, Diego Ardissino

**Affiliations:** ^1^Imperial College London, London, United Kingdom; ^2^Duke Clinical Research Institute, Durham, NC, United States; ^3^South Australian Health and Medical Research Institute (SAHMRI), Adelaide, SA, Australia; ^4^Clinical and Epidemiological Research Unit, University Hospital of Parma, Parma, Italy; ^5^Division of Cardiology, Azienda Ospedaliero-Universitaria di Parma, Parma, Italy; ^6^Department of Genetics, Evolution and Environment, UCL Genetics Institute, University College, London, United Kingdom; ^7^Division of Cardiology, Ospedale San Camillo, Rome, Italy; ^8^Division of Cardiology, Fondazione IRCCS, Policlinico San Matteo, Pavia, Italy; ^9^Associazione per lo Studio della Trombosi in Cardiologia, Pavia, Italy; ^10^Cardiothoracic Department, University Hospital “Santa Maria della Miserciordia”, Udine, Italy; ^11^Division of Cardiology, Azienda Ospedaliera, Ospedale Niguarda Cà Granda, Milan, Italy; ^12^ICCU, Intensive and Interventional Cardiology, San Filippo Neri Hospital, Rome, Italy; ^13^Centre for Biostatistics, School of Health Sciences, University of Manchester, Manchester, United Kingdom; ^14^Department of Cardiology, Antonio Perrino Hospital, Azienda Sanitaria Locale di Brindisi, Brindisi, Italy; ^15^Department of Cardiology, Sant'Anna Hospital, Como, Italy; ^16^Division of Cardiology, Istituti Ospitalieri, Cremona, Italy; ^17^Hospital Universitario de Toledo, Servizio di Radiologia, Toledo, Spain; ^18^Division of Cardiology, San Luigi Gonzaga University Hospital, Turin, Italy; ^19^Division of Cardiology, Ospedale di Legnano, Legnano, Italy; ^20^Department of Biomedical Sciences, Humanitas Clinical and Research Center IRCCS, Humanitas University, Milan, Italy; ^21^Division of Cardiology, Cardiocentro Ticino, Lugano, Switzerland; ^22^University Division of Clinical Cardiology, AORN Sant'Anna e San Sebastiano, Caserta, Department of Translational Medical Sciences, Luigi Vanvitelli University of Campania, Naples, Italy

**Keywords:** gender, long term outcomes, baseline risk differences, myocardial infarction, young

## Abstract

**Importance:**

There is growing awareness of sex-related differences in cardiovascular risk profiles, but less is known about whether these extend to pre-menopausal females experiencing an early-onset myocardial infarction (MI), who may benefit from the protective effects of estrogen exposure.

**Methods:**

A nationwide study involving 125 Italian Coronary Care Units recruited 2,000 patients between 1998 and 2002 hospitalized for a type I myocardial infarction before the age of 45 years (male, *n* = 1,778 (88.9%). Patients were followed up for a median of 19.9 years (IQR 18.1–22.6). The primary composite endpoint was the occurrence of cardiovascular death, non-fatal myocardial re-infarction or non-fatal stroke, and the secondary endpoint of hospitalization for revascularisation by means of a percutaneous coronary intervention (PCI) or coronary artery bypass surgery (CABG).

**Results:**

ST-elevation MI was the most frequent presentation among both men and women (85.1 vs. 87.4%, p = ns), but the men had a greater baseline coronary atherosclerotic burden (median Duke Coronary Artery Disease Index: 48 vs. 23; median Syntax score 9 vs. 7; both *p* < 0.001). The primary composite endpoint occurred less frequently among women (25.7% vs. 37.0%; adjusted hazard ratio: 0.69, 95% CI 0.52–0.91; *p* = 0.01) despite being less likely to receive treatment with most secondary prevention medications during follow up.

**Conclusions:**

There are significant sex-related differences in baseline risk factors and outcomes among patients with early-onset MI: women present with a lower atherosclerotic disease burden and, although they are less frequently prescribed secondary prevention measures, experience better long-term outcomes.

**Trial Registration:**

4272/98 Ospedale Niguarda, Ca' Granda 03/09/1998.

## Introduction

Cardiovascular disease is a major cause of morbidity and mortality in both men and women. However, sex-related differences in the presentation, treatment and outcomes of myocardial infarction (MI) have been extensively described with women more likely to be older, have greater risk factor burden and more varied symptoms at presentation. Women also tend to have worse short- and long-term outcomes which may be compounded by consistent observations that women are less likely to receive secondary prevention medications and achieve risk factor control (1–10). These differences have so far mostly been addressed in relation to the general population of MI patients, and few studies so far have characterized sex-related differences in long-term outcomes after an early-onset MI.

Early-onset MI is a major health burden because, although the rates of MI as a whole have decreased, those with early-onset MI have remained the same. Furthermore, the affected patients are at high risk of a recurrence and as many as one-third will experience at least one other major cardiovascular event within 20 years of the first (11, 12). Characterizing the long-term outcomes of early-onset MI by sex is also of importance given the modulating effect of estrogen exposure on cardiovascular risk in females.

The Italian Genetic Study on Early-onset Myocardial Infarction is a multi-center, nationwide study designed to evaluate risk factors and outcomes of patients experiencing an MI before the age of 45 years. We aimed to explore sex-related differences in the presentation, treatment and long-term clinical outcomes of patients who suffer an early-onset MI.

## Methods

The Italian Genetic Study on Early-onset Myocardial Infarction is a nation-wide project aimed at investigating the genetics of susceptibility to MI. It consists of an initial case-control study followed by a prospective cohort study designed to describe the long-term outcomes of the cases (13).

### Patient Population

The Italian Genetic Study on Early-onset Myocardial Infarction enrolled consecutive patients from 125 Italian Coronary Care Units between 1998 and 2002. Patients under the age of 45 years who had undergone coronary angiography during hospitalization for their index MI were eligible for participation. The initial diagnosis of the index event was based on a combination of: (1) symptoms suggesting myocardial ischemia; (2) electrocardiographic changes consistent with acute myocardial ischemia; and (3) an increase in CK-MB levels to more than twice the upper limit of normal with a typical rise and fall pattern.

### Study Protocol

The protocol was approved by the Ethics Committee of the coordinating center, and all of the study patients gave their written informed consent to enrolment. A standardized case report form (CRF) was completed for every patient suitable for enrolment in order to collect detailed information concerning their cardiovascular risk factors, lifestyle habits and medications, the history of any cardiovascular diseases affecting each member of their families (including first- and second-degree relatives), and the patients' own clinical, demographic and treatment characteristics. The definitions of all of these factors are described in detail in Supplementary Material 1.

The patients were followed up in order to track subsequent hospital admissions for cardiovascular or ischemic cerebrovascular events. The occurrence of these endpoints was assessed by means of scheduled outpatient visits and standardized telephone follow up. Every attempt was made to contact all of the patients or their primary care physicians; if this was not possible, a member of their family was contacted. If an event was reported by a patient, relative or general practitioner, the related medical record(s) were obtained.

### Coronary Angiography

All of the patients underwent coronary angiography at the time of the index event. Coronary arteries were considered normal in the absence of angiographic stenosis: a narrowing of <70% (or <50% in the case of the left main coronary artery) was considered non-significant coronary artery stenosis, and a narrowing of > 70% (or >50% in the case of the left main coronary artery) was considered significant coronary artery stenosis. Single-vessel disease was recorded when a significant stenosis was identified in only one major coronary artery, and multi-vessel disease when a significant stenosis was identified in two or more major epicardial coronary arteries. Left main coronary artery disease was considered multi-vessel disease. The total extent and complexity of the angiographic coronary artery disease burden was quantified using the Duke Coronary Artery Disease Index (14) and the Syntax score (15).

### Endpoints

The primary composite endpoint was the occurrence of cardiovascular death, the re-occurrence of non-fatal MI, or the occurrence of a non-fatal ischemic stroke. All of the reported deaths were verified on the basis of death certificates specifying the cause(s) of death, and each reported event was investigated by means of source data verification. The outcome of cardiovascular death was defined as any death attributed to a cardiovascular cause on the death certificate. The re-occurrence of MI was defined as any hospitalization during follow-up ending with a discharge diagnosis of MI. The occurrence of stroke was defined as hospitalization ending with a discharge diagnosis of ischemic stroke.

The secondary study endpoint was any hospital admission that involved revascularisation by means of a percutaneous coronary intervention (PCI) or coronary artery by-pass graft (CABG) but did not end in a diagnosis of MI.

All of the documented events with verified source data were adjudicated by a dedicated Clinical Events Committee consisting of two cardiologists; in the case disagreement, the opinion of a third cardiologist was sought.

### Data Management

The study's clinical data were recorded using an off-line electronic CRF (e-CRF). The demographic, clinical, angiographic and treatment characteristics of all patients were collected at the time of the index event and recorded in the e-CRF along with events ascertained during follow up. Visual Basic for Applications (VBA) and SQL scripts were used to manage the database and make preliminary data checks during data entry. In the case of missing data, queries were generated and sent to study sites for resolution.

### Statistical Analysis

The study cohort was divided into females and males, and descriptive statistics were used to compare the baseline demographic and clinical characteristics of the two groups. Univariable logistic regression analysis was used to evaluate the relationship between covariates and outcomes. Variables included age, weight, height, body mass index, type of acute coronary syndrome at the time of the index event (non-ST elevation MI [NSTEMI] vs. ST elevation MI [STEMI]), family history of coronary artery disease (CAD), hypertension, dyslipidemia, diabetes, alcohol consumption, smoking, physical activity, cocaine use, estrogen replacement therapy, previous thromboembolic events, ejection fraction, pattern and extent of angiographic disease burden (Duke Coronary Artery Disease Index, Syntax score, presence of multivessel disease), presence and mode of coronary artery revascularisation, and pharmacological treatments. The variables with a *p*-value of <0.2 were selected for inclusion in a comprehensive logistic regression model of long-term outcomes that was subsequently simplified by means of the step-wise backward selection of independent predictors, which were then used to make an adjusted proportional hazard analysis of long-term outcomes.

The time to the first endpoint and the time to all endpoints by type were analyzed for their dependence on their putative predictors using Cox proportional hazard models. The proportional hazard assumption for the Cox regression model was confirmed by means of Schoenfeld's residuals test. The cumulative incidence of primary endpoints during follow-up was graphically depicted using Aalen-Johansen curves, and the significance of the differences between the hazard sub-distributions was tested using the Fine-Gray model. An Andersen-Gill intensity model analysis was not pre-specified, but was made in order to account for the repeated occurrence of all of the components of the primary and secondary endpoints during the study period using a time-dependent model.

All of the tests were two-sided, and a *p*-value of 0.05 was considered statistically significant. Given the consecutive nature of the enrolment, there was an uneven distribution of patients across sexes. For this reason, a *post-hoc* power calculation was performed based based on the number of patients by sex (222 females, 1,778 males), and incidence of primary events (25.7% vs. 37.0%), and alpha = 0.05. This yielded a power of 93.3%. The statistical analyses were made using R Statistical software, version 3.6.0 (R Foundation for Statistical Computing, Vienna, Austria).

## Results

The Italian Genetic Study on Early-onset Myocardial Infarction enrolled a total of 2,000 patients who had experienced a first MI before they were 45 years old: 1,778 men (88.9%) and 222 women (11.1%). The baseline demographic, clinical, angiographic and treatment characteristics of the study population are shown in Table 1. Median age at the time of presentation was 41 years (interquartile range [IQR] 37–43), and was similar among the men (41, IQR 37–43) and women (40, IQR 36–43).

**Table 1:** Baseline demographic, clinical, angiographic and treatment characteristics of the study population by sex.

	**All (*n* = 2,000)**	**Females (*n* = 222)**	**Males (*n* = 1,778)**	***P*-value**
**DEMOGRAPHIC CHARACTERISTICS**				
Median age, years (IQR)	41(37–43)	40(36–43)	41(37–43)	ns
Median weight, kg (IQR)	78(70–88)	60(53–68)	80(72–90)	<0.001
Median height, cm (IQR)	172(168–177)	160(156–165)	173(169–178)	<0.001
Median BMI, kg/cm^2^ (IQR)	26.3(24.1–29.1)	23.2(21.5–25.9)	26.6(24.3–28.4)	ns
**CLINICAL CHARACTERISTICS**				
Index event STEMI	1,707(85.4%)	194(87.4%)	1,513(85.1%)	ns
Index event NSTEMI	293(14.6%)	28(12.6%)	265(14.9%)	ns
Family history of CAD	1,627(81.3%)	183(82.4%)	1,444(81.2%)	ns
Hypertension	536 (26.8%)	59(26.6%)	477(26.8%)	ns
Dyslipidemia	1,184/1,936 (61.2%)	101/218(46.3%)	1,083/1,718(63%)	<0.001
Diabetes	154(8.0%)	13(6.0%)	141(8.0%)	ns
Alcohol consumption	1,225/1,994(61.4%)	60(27.0%)	1,165/1,772(65.8%)	<0.001
Smokers	920(46.0%)	95(42.8%)	825(46.4%)	<0.001
**Exercise**				
None	1,067/1,991(53.6%)	149(67.1%)	917/1,769(51.8%)	<0.001
Moderate	418/1,991(21%)	25(11.3%)	393/1,769(22.2%)	
Intense	507/1,991(25.4%)	48(21.6%)	459/1,769(26%)	
Cocaine use	57(2.9%)	4(1.8%)	53(3.0%)	ns
Estrogen therapy	N/A	97/218(44.5%)	N/A	N/A
Previous thromboembolism	317(15.9%)	34(15.3%)	283(15.9%)	ns
**CORONARY ANGIOGRAPHY**				
**Coronary artery disease**				
Normal	264(13.2%)	61(27.5%)	203(11.4%)	<0.001
Non-significant	90(4.5%)	20(9.0%)	70(4.0%)	
Single-vessel disease	896(44.8%)	104(46.8%)	792(44.5%)	
Multi-vessel disease	750(37.5%)	37(16.7%)	713(40.1%)	
Median Duke Coronary Artery Disease Index (IQR)	48(23–56)	23(0–48)	48(23–56)	<0.001
Median Syntax score, (IQR)	9(4–15)	7(0–11)	9(5–15)	<0.001
Spontaneous coronary artery dissection	24(1.2%)	12(5.4%)	12(0.7%)	<0.001
Revascularisation at time of index event	839(42%)	82(36.9%)	757(42.5%)	ns
PCI at time of index event	669(33.5%)	72(32.4%)	597(33.5%)	ns
CABG at time of index event	170(8.5%)	10(4.5%)	160(9%)	0.02
**TREATMENT** ^*^				
Beta-blocker	1,629(81.4%)	166(74.8%)	1,463(82.3%)	<0.02
Aspirin	1,860(93.0%)	192(86.5%)	1,668(93.8%)	<0.001
P2Y12 inhibitor	1,009(50.5%)	96(43.2%)	913(51.3%)	<0.02
ACE-inhibitor or ARB	865(43.3%)	73(32.9%)	792(44.5%)	<0.001
Statin	1,949(97.5%)	214(96.4%)	1,735(97.6%)	ns

*The denominator is shown in the case of missing data*.

* *Treatment received at any stage during follow-up. ACE, angiotensin-converting enzyme; ARB, angiotensin receptor blockade. CABG, coronary artery bypass graft; IQR, interquartile range; CVD, cardiovascular disease; NSTEMI, non-ST segment elevation myocardial infarction; PCI, percutaneous coronary intervention. STEMI, ST segment elevation myocardial infarction*.

There were no significant sex-related differences in the frequency of a positive family history of CAD, the presence of hypertension or diabetes, or the occurrence of a previous thromboembolic event. A significantly higher proportion of the men were smokers (46.4% vs. 42.8%; *p* < 0.001) and consumed alcohol (65.8% vs. 27.0%; *p* < 0.001). Dyslipidemia was more frequent in men (63% vs. 46.3%; *p* < 0.001), as was self-reported exercise (*p* < 0.001).

The majority of the cohort (85.4%) presented with STEMI, and there was no sex-related difference in the proportion presenting with STEMI compared to NSTEMI. Women were more than twice as likely to have normal or non-significant coronary artery stenoses at the time of angiography, and much more likely to have spontaneous coronary artery dissection (SCAD) (5.4% vs. 0.7%, *p* < 0.001). In contrast, men were significantly more likely to have multi-vessel disease (40.1% vs. 16.7%, *p* < 0.001). The greater extent and complexity of the angiographic coronary artery disease burden among the men was reflected by their higher median Duke Coronary Artery Disease Index (48 vs. 23, *p* < 0.001) and Syntax score (9 vs. 7, *p* < 0.001).

During hospitalization for the index event, rates of PCI were similar among men and women (33.5% vs. 32.4%; *p* = ns) however a greater proportion of men (9.0% vs. 4.5%; *p* = 0.02) underwent CABG.

At discharge, men were significantly more likely to be prescribed beta-blockers (82.3% vs. 74.8%; *p* = 0.02), aspirin (93.8% vs. 86.5%; *p* < 0.001), P2Y12 inhibitors (51.3% vs. 43.2%, *p* = 0.02), and ACE-inhibitors or ARBs (44.5% vs. 32.9%; *p* < 0.001). Statin treatment was similar in the two groups (97.5% vs. 96.4%, *p* = ns).

### Primary and Secondary Endpoints

**Figure 1:**
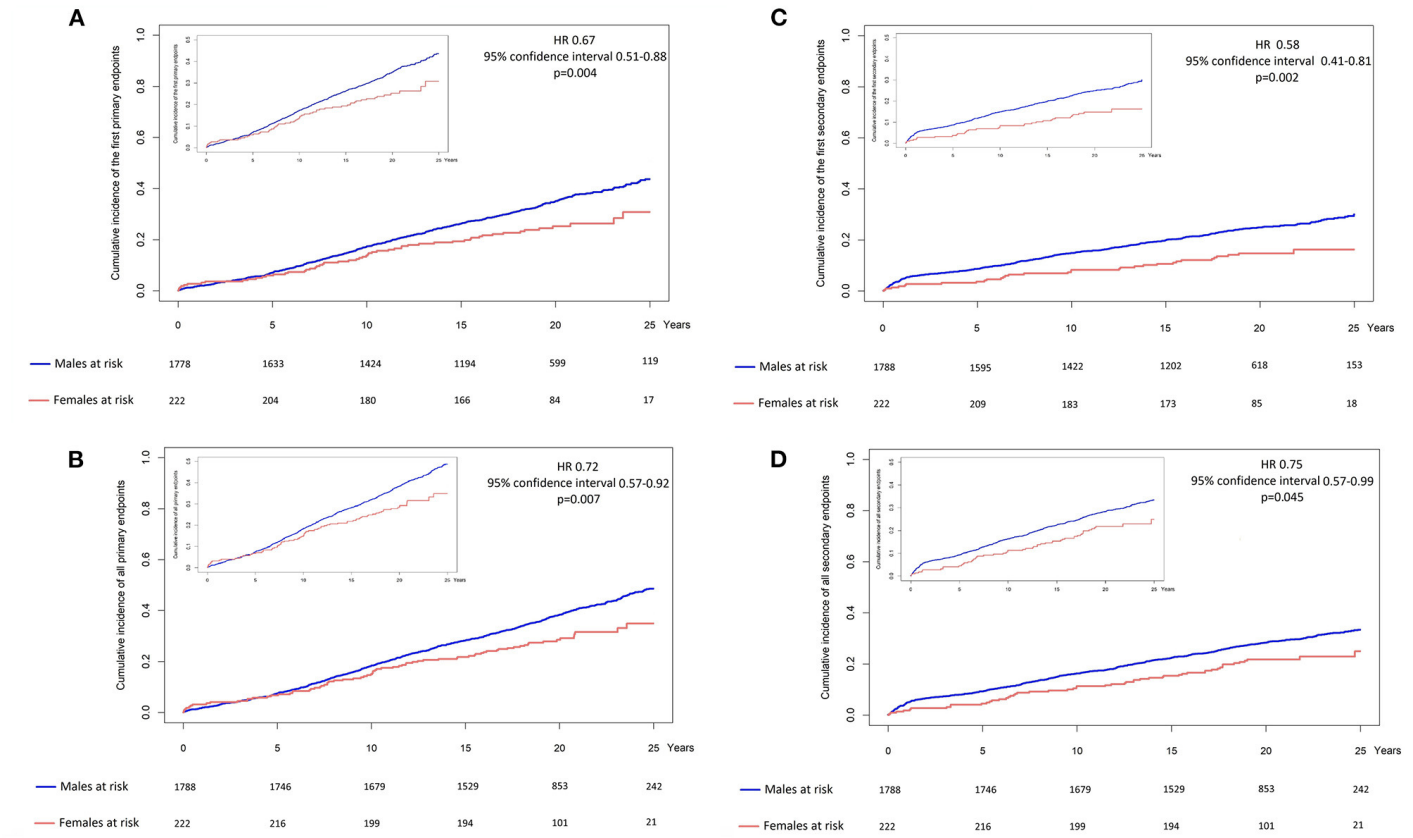
**(A)** Cumulative incidence of the first primary endpoint by sex. **(B)** Cumulative incidence of the first secondary endpoint by sex. **(C)** Cumulative incidence of all primary endpoints by sex. **(D)** Cumulative incidence of all secondary endpoints by sex.

The patients were followed up for a median 19.9 years (IQR 18.1–22.6) or 39,535 person-years. Follow-up was complete in 1,984 patients (99.2%), and vital status was ascertained in 1,988 (99.4%). A primary endpoint was experienced by 714 patients (36%): 153 died of cardiovascular causes, 479 experienced a recurrent non-fatal MI, and 82 an acute ischemic stroke. Table 2 and Figures 1A,B show the number and rate of first occurrences of the primary and secondary endpoints by sex.

**Table 2:** First occurrence of primary and secondary endpoints during follow-up by sex.

**Events**	**All patients (*n =* 1,984)**	**Females (*n =* 218)**	**Males (*n =* 1,766)**	**Adjusted hazard ratio (F:M)**	**95% confidence interval**	***P*-value**
**Primary endpoints**						
Primary composite endpoint	714 (35.9%)	57 (26.1%)	657 (37.2%)	0.69	0.52–0.91	0.01
Cardiovascular death	153 (7.7%)	9 (4.1%)	144 (8.1%)	0.57	0.30–1.08	0.082
Non-fatal myocardial re-infarction	479 (24.1%)	31 (14.2%)	448 (25.4%)	0.53	0.37–0.77	<0.001
Non-fatal ischemic stroke	82 (4.1%)	17 (7.8%)	65 (3.7%)	2.02	1.17–3.49	0.012
**Secondary endpoints**						
Hospitalization for revascularisation	484 (24.4%)	34 (15.6%)	450 (25.5%)	0.60	0.41–0.86	0.006
By means of PCI	301 (15.2%)	21 (9.6%)	280 (15.9%)	0.59	0.38–0.91	0.018
By means of CABG	183 (9.2%)	13 (6.0%)	170 (9.6%)	0.61	0.35–1.07	0.084

During follow-up, the primary composite endpoint occurred less frequently among the women (26.1% vs. 37.2%; adjusted hazard ratio (aHR) 0.69, 95% CI 0.52–0.91; *p* = 0.01). Cardiovascular death was also less frequent among women although this did not reach statistical significance (4.1% vs. 8.1%, aHR 0.57, 95% CI 0.30–1.08, *p* = 0.082). Non-fatal myocardial re-infarction was significantly less frequent among the women (14.2% vs. 25.4%; aHR 0.53, 95% CI 0.37–0.77; *p* < 0.001), although women were more likely to experience a non-fatal ischemic stroke (7.8% vs. 3.7%; aHR 2.02, 95%CI 1.17–3.49; *p* = 0.012).

The occurrence of hospitalization for revascularisation was significantly less frequent among the women (15.6% vs. 25.5%; aHR 0.60, 95%CI 0.41–0.86; *p* = 0.006), driven largely by statistically less frequent PCI (9.6% vs. 15.9%; aHR 0.59, 95% CI 0.38–0.91; *p* = 0.018) and directionally consistent lower rates CABG (6.0% vs. 9.6%, aHR 0.61, 95% CI 0.35–1.07, *p* = 0.084).

The study population as a whole experienced a total of 973 composite primary events including repeated events. Table 3 and Figures 1C,D show the number of all occurrences of primary and secondary endpoints by sex. The total number of primary endpoints was significantly lower among the women (aHR 0.75; 95% CI 0.59–0.96; *p* = 0.02). With respect to the individual components of the composite, women were less likely to experience cardiovascular death (aHR 0.57, 95% CI 0.35–0.95; *p* = 0.032) or non-fatal myocardial re-infarction (aHR 0.62, 95% CI 0.45–0.86; *p* = 0.004). While not reaching statistical significance, there was a non-significant trend toward higher rates of ischaemic stroke in women (aHR1.6; 95% CI 0.97–2.7; *p* = ns) and a trend toward lower rates of admission for further revascularization (aHR 0.78; 95% CI 0.58–1.04; *p* = ns).

**Table 3:** Total occurrence of primary and secondary endpoints during follow-up by sex.

**Events**	**All patients (*n =* 1,984)**	**Females (*n =* 218)**	**Males (*n =* 1,766)**	**Adjusted hazard ratio (F:M)**	**95% confidence interval**	***P*-value**
**Primary endpoints**						
Primary composite endpoint	973	75	898	0.75	0.59–0.96	0.02
Cardiovascular death	246	16	230	0.57	0.35–0.95	0.032
Non-fatal myocardial re-infarction	617	41	576	0.62	0.45–0.86	0.004
Non-fatal ischemic stroke	110	18	92	1.6	0.97–2.7	ns
**Secondary endpoints**						
Hospitalization for revascularisation	671	55	616	0.78	0.58–1.04	ns
By means of PCI	464	39	425	0.77	0.56–1.07	ns
By means of CABG	207	16	191	0.71	0.42–1.18	ns

### Outcome Predictors

Table 4 shows the factors associated with experiencing the primary endpoint during follow-up. Multiple logistic regression analysis identified only two variables that were independently associated among women; hypertension (OR 2.56; 95% CI 1.01–6.58; *p* = 0.05) and a positive family history of CAD (OR 8.53, 95% CI 1.58–159, *p* = 0.04). Among men there were six variables with independent associations including the Duke Coronary Artery Disease Index (OR 1.01; 95% CI 1–1.01; *p* = 0.02), hypertension (OR 1.34; 95% CI 1.01–1.78; *p* = 0.04), dyslipidemia (OR 1.41; 95% CI 1.1–1.8; *p* = 0.01), diabetes (OR 2.1; 95% CI 1.33–3.36 *p* = 0.002), and previous thromboembolic event (OR 2.45; 95% CI 1.71–3.52; *p* < 0.001). An inverse association with the ejection fraction was noted (OR 0.98 95% CI 0.97–0.99 *p* = 0.006). The occurrence of a secondary endpoint was independently associated with the Duke Coronary Artery Disease Index (OR 1.02; 95% CI 1.0–1.04; *P* < 0.02) and diabetes (OR 6.17; 95% CI 1.2–36.5; *p* = 0.03) among women, and alcohol consumption (OR 0.72; 95% CI 0.55–0.93; *p* = 0.01), the Duke Coronary Artery Disease Index (OR 1.01; 95% CI 1.01–1.02; *p* < 0.001), a previous thromboembolic event (OR 1.64; 95% CI 1.15–2.34; *p* = 0.007), and diabetes (OR 2.88; 95% CI 1.81–4.67; *p* < 0.001) among men.

**Table 4:** Independent predictors of recurrence of primary and secondary composite endpoints.

**Explanatory variable**	**Adjusted odds ratio**	**95% confidence interval**	**P-value**
**PRIMARY COMPOSITE ENDPOINT**			
**Females**			
Hypertension	2.56	1.01–6.58	0.05
Family history of CAD	8.53	1.58–159	0.04
**Males**			
Ejection fraction	0.98	0.97–0.99	0.006
Duke Coronary Artery Disease Index	1.01	1.0–1.01	0.02
Hypertension	1.34	1.01–1.78	0.04
Dyslipidemia	1.41	1.1–1.8	0.01
Diabetes	2.1	1.33–3.36	0.002
Previous thromboembolic event	2.45	1.71–3.52	<0.001
**SECONDARY COMPOSITE ENDPOINT**			
**Females**			
Duke Coronary Artery Disease Index	1.02	1.0–1.04	0.02
Diabetes	6.17	1.2–36.5	0.03
**Males**			
Alcohol consumption	0.72	0.55–0.93	0.01
Duke Coronary Artery Disease Index	1.01	1.01–1.02	<0.001
Previous thromboembolic event	1.64	1.15–2.34	0.007
Diabetes	2.88	1.81–4.67	<0.001

## Discussion

This long-term follow up study of a large population of early-onset myocardial infarction patients reveals that, unlike in the general population, women experienced significantly better long-term outcomes than men. It is known that early-onset MI generally has a poor prognosis, with estimates that as many as one-third of affected patients experience death or the recurrence of a major cardiovascular event during the 20 years after the initial MI (10–12) Furthermore, the detailed angiographic description, robust adjudication of outcomes and rigorous event ascertainment with <1% loss to follow up over a median of almost 20 years represent key strengths of this study.

An established body of evidence suggests that, among the general MI population of all ages, women have worse in-hospital (8) and long-term outcomes after an acute coronary syndrome, whereas there is little and conflicting evidence concerning young MI cohorts. Seminal studies of administrative data relating to patients aged <60 years and treated in the early 1990s found higher in-hospital (16) and 2-year mortality (17) among women, but others did not (18, 19). Similarly, more recent prospective registries that have deliberately over-enrolled younger female patients show no evidence of sex-related differences in major adverse cardiovascular events after 1 year (10). After multivariable adjustment for traditional risk factors, lipid profiles, angiographic disease and medications prescribed at any point during follow up, the women in our cohort were 30% less likely to experience an adverse cardiovascular outcome during follow-up than the men. Furthermore, although they were more likely to experience a non-fatal stroke, they were half as likely to sustain a non-fatal MIs, and were also less likely to die of cardiovascular causes.

There are a number of factors that may have contributed to these findings. Firstly, the effect of a number of known contributors to worse outcomes among women may have been attenuated by our study's enrolment process: only enrolling patients who underwent angiography may have reduced the possibility of a delayed or “atypical” clinical presentation leading to under-investigation, a missed diagnosis and under-treatment, which are known to be more frequent among women (20, 21). Secondly, possible improvements in the care of women over the last 20 years due to a greater awareness and recognition of heart disease in women may have led to more timely investigation and diagnosis. Finally, there are important sex-related differences in the etiology of the index events. The women in the study had a generally similar risk factor profile to that of the men, but their burden of angiography-detected atherosclerotic disease was significantly less. Although MI with no obstructive coronary artery (MINOCA) had not been identified as a separate clinical entity at the time of our cohort's angiography for their index event, we observed more women with MINOCAs and spontaneous coronary artery dissections, and the cohort may feasibly also have suffered more coronary artery vasospasm and takotsubo cardiomyopathy—all of which are more frequent in women and have a favorable impact on prognosis insofar as they are usually stand-alone clinical events. Furthermore, it is known that younger pre-menopausal women have more diffuse, non-obstructive atherosclerosis (22, 23) and higher rates of microvascular dysfunction (24, 25) than older women, and so endogenous hormone levels may have contributed to more benign index events.

One of the limitations of this study is that it does not include patients who were troponin negative or did not undergo angiography, which means that its findings cannot be generalized to all patients with an acute coronary syndrome. Furthermore, a number of advances in MI care have occurred since these patients were treated (drug-eluting stents, widespread P2Y12 inhibition, potent low-density lipoprotein lowering) and so the observed recurrent event rates may not reflect outcomes in contemporary clinical practice.

## Conclusions

In this very long-term study of the largest series of MI patients aged <45 years who underwent coronary angiography for their index event, almost one-third of patients experienced an adverse cardiovascular outcome over a period of 20 years. The overall adjusted cardiovascular event rates were almost one-third lower in women despite the fact that they received fewer preventive treatments when discharged, although it should be noted that men had a greater burden of angiography-detected atherosclerotic disease at the time of the index event.

## Data Availability Statement

The raw data supporting the conclusions of this article will be made available by the authors, without undue reservation.

## Ethics Statement

The studies involving human participants were reviewed and approved by Comitato Etico Area Vasta Emilia Nord. The patients/participants provided their written informed consent to participate in this study.

## Author Contributions

All authors listed have made a substantial, direct, and intellectual contribution to the work and approved it for publication.

## Funding

The Italian Genetic Study on Early-onset Myocardial Infarction was funded by the *Associazione per lo Studio della Trombosi in Cardiologia*. The follow-up of the patients was funded by the Emilia-Romagna Region: Programma Regione Università Cardiovascular Genetics: from bench to bedside, CUP E35E09000880002.

## Conflict of Interest

The authors declare that the research was conducted in the absence of any commercial or financial relationships that could be construed as a potential conflict of interest.

## Publisher's Note

All claims expressed in this article are solely those of the authors and do not necessarily represent those of their affiliated organizations, or those of the publisher, the editors and the reviewers. Any product that may be evaluated in this article, or claim that may be made by its manufacturer, is not guaranteed or endorsed by the publisher.
