# Supplementary material for: Sex-Related Differences in Long-Term Outcomes After Early-Onset Myocardial Infarction
Source: Front Cardiovasc Med. 2022 Jul 4;9:863811. doi: 10.3389/fcvm.2022.863811 (PMC9289186; doi:10.3389/fcvm.2022.863811)
Supplement: Supplementary file 1 [file Data_Sheet_1.docx]

**SUPPLEMENTARY MATERIAL 1: CLINICAL, DEMOGRAPHIC AND TREATMENT CHARACTERISTICS.**

**Definitions**

**Index event: acute myocardial infarction**

Acute myocardial infarction was defined as resting chest pain lasting for 30 minutes accompanied by ECG changes indicative of ischemia; and was confirmed by the presence of levels of total creatinine kinase or its MB fraction of more than twice the upper normal limit and following a typical rise and fall pattern.

Acute myocardial infarction with ST segment elevation (STEMI) was defined as a new ST elevation at the J point in two contiguous leads of ≥0.1 mV in all leads other than leads V2-V3, in which the elevation had to be ≥0.2 mV in men aged ≥ 40 years (≥0.25 mV in men aged <40 years) or ≥0.15 mV in women.

All cases of acute myocardial infarction that did not satisfy the electrocardiographic criteria of STEMI were considered to be acute myocardial infarction without ST segment elevation (NSTEMI).

**Family history of cardiovascular disease**

A positive family history was defined as the presence of at least one first-degree relative (parent, offspring, or sibling) who had developed coronary artery disease before the age of 55 years (men) or 65 years (women).

**Hypertension**

The subjects were considered to be hypertensive if they had been diagnosed as such by a physician or if they were taking anti-hypertensive medication.

**Obesity**

Body mass index (BMI) values were defined as normal (18.5-25 kg/m^2^), pre-obese (25-30 kg/m^2^) or obese (30-35 kg/m^2^), with the last including WHO classes I, II and III; underweight subjects were excluded from the analysis because of their small number.

**Smoking habits**

The subjects were classified on the basis of self-reports as current smokers (those who reported smoking regularly during the three years preceding the myocardial infarction), former smokers (those who had smoked regularly for at least three years but not during the year preceding the infarction), or never smokers (those who had never smoked regularly or had not smoked regularly for three years. Never and former smokers were put into the single category of non-smokers.

**Diabetes**

The subjects were considered to be diabetic if they had ever been diagnosed as having type I or II diabetes by a physician.

**Hypercholesterolemia**

Hypercholesterolemia was defined as a fasting total serum cholesterol level of 200 mg/dL (5.2 mmol/L) or taking anti-hypercholesterolemic medications.

**Cocaine use**

Cocaine use was classified as chronic, occasional, or absent at the time of the index infarction or enrolment.

**Exercise**

Exercise was considered habitual (category 2) if the subjects engaged in moderately intense exercise for 30 minutes every day or vigorous exercise for 45 minutes twice a week or 20 minutes three times a week. Any other level of physical activity was considered occasional (category 1), and no exercise as category 0.

**Alcohol consumption**

Alcohol consumption was classified on the basis of self-reports as moderate (10-30 g of ethanol/day) or high (>30 g of ethanol/day). In the statistical analysis, it was considered a dichotomous (yes/no) variable, with moderate and high consumers being put in the same category.

**Estrogen therapy**

Estrogen therapy was defined as the current (at the time of index event) or previous continuous intake of estrogen for clinical indications (hormone replacement therapy in early menopause, endocrinological diseases and dermatological diseases)**.** Estrogen-progestin contraceptive pills were not considered in this category.

**Previous thromboembolic events**

A previous thromboembolic event was defined as any previous venous or arterial cardiovascular event other than acute myocardial infarction or acute ischemic or hemorrhagic stroke (e.g. acute pulmonary embolism, acute deep vein thrombosis, acute limb ischemia, acute ischemia of the gastrointestinal tract, acute ischemia of the kidneys, central retinal vein occlusion).

**Treatment**

All medical treatments received at any stage during follow-up were recorded and considered in the analysis.

**SUPPLEMENTARY MATERIAL 2: ANGIOGRAPHIC CHARACTERISTICS**

**Definitions**

**Normal coronary arteries**

The absence of any narrowing in coronary diameter.

**Non-significant coronary arteries stenosis**

A narrowing of <70% (<50% in the case of the left main coronary artery).

Significant coronary arteries stenosis

A narrowing of >70% (>50% in the case of the left main coronary artery)

**Spontaneous coronary artery dissection**

This was defined as an epicardial coronary artery dissection not associated with atherosclerosis or trauma, and not iatrogenic.

**Single-vessel disease**

This was recorded when a significant stenosis was identified in only one major coronary artery

**Multi-vessel disease**

This was recorded when a significant stenosis was identified in two or more major coronary arteries

**Syntax Score**

The SYNTAX (SYNergy between PCI with TAXUS and Cardiac Surgery) score was created to grade the angiographic complexity of coronary artery disease in patients with left main or multivessel disease. Each lesion with a diameter stenosis ≥50% in vessels at least 1.5mm in calibre are scored. Each lesion can involve ≥1 diseased segments. Serial stenoses less than 3 vessel reference diameters apart are scored as one lesion, however stenoses at a greater distance from each other (>3 vessel reference diameters), are considered separate lesions.

**Duke Coronary Artery Disease Index**

The Duke Coronary Artery Disease Index was introduced by Mark *et al*. in 1995^1^. It is used to assess the clinical significance and prognostic weight of coronary artery disease based on angiographic relief. A stenosis of >50% is classified as clinically significant. The other variables are the number of vessels involved and the location of obstructive lesions (the greatest prognostic weight for left main and proximal left anterior descending coronary artery involvement). The prognostic weight of coronary artery disease is expressed using a scale ranging from 0 (<50% stenosis) to 100 (>70% stenosis in the left main coronary artery).

**References**

*1. Mark DB, Nelson CL, Califf RM, et al. Continuing evolution of therapy for coronary artery disease: initial results from the era of coronary angioplasty. Circulation 1994;89:2015-2025*

*2 Serruys PW, Onuma Y, Garg S, et al* [*Assessment of the SYNTAX score in the Syntax study.*](https://www.ncbi.nlm.nih.gov/pubmed/19577983) *EuroIntervention. 2009 May;5(1):50-6*

**SUPPLEMENTARY MATERIAL 3: END-POINT DEFINITIONS**

**End-points**

The primary composite endpoint was defined as the occurrence of cardiovascular death, the re-occurrence of non-fatal MI, or the occurrence of a non-fatal ischemic stroke. The secondary endpoint was defined as any hospital admission that involved revascularisation by means of a percutaneous coronary intervention (PCI) or a coronary artery bypass graft (CABG), but did not end in a diagnosis of MI.

**Cardiovascular death and sudden cardiac death**

All deaths reported were recorded and adjudicated on the basis of death certificates. Cardiovascular death was defined as death due to cardiovascular causes, and included sudden cardiac death, death due to an acute myocardial infarction, death due to heart failure, death due to a cerebrovascular event, death due to other cardiovascular causes (e.g. pulmonary embolism, aortic disease, cardiovascular intervention)

Sudden cardiac death refers to a death that occurs unexpectedly not following an acute myocardial infarction:

- Witnessed death occurring without new or worsening symptoms;
- Witnessed death occurring within 60 minutes of the onset of new or worsening cardiac symptoms, unless the symptoms suggest acute myocardial infarction;
- Witnessed death attributed to an identified arrhythmia (e.g. captured on an ECG recording, witnessed on a monitor, or unwitnessed but found upon the review of an implantable cardioverter-defibrillator);
- Death after unsuccessful resuscitation from cardiac arrest (e.g. implantable cardioverter-defibrillator unresponsive sudden cardiac death, pulseless electrical activity arrest);
- Death after successful resuscitation from cardiac arrest without the identification of a specific cardiac or non-cardiac etiology;
- Unwitnessed death in a subject seen alive and clinically stable ≤24 hours before being found dead without any evidence supporting a specific non-cardiovascular cause of death (if available, information regarding the patient’s clinical status preceding death should be provided).

**Non-fatal myocardial infarction**

Myocardial infarction was defined in accordance with the universal definition.^1^ Any one of the following meets the criteria for a diagnosis of myocardial infarction:

1. Detection of a rise and/or fall in the level of cardiac biomarkers (preferably troponin), with at least one value above the 99^th^ percentile of the upper reference limit (URL). together with at least one of the following signs of myocardial ischemia:

• Symptoms of ischemia;

• ECG changes indicative of new ischemia (new ST-T changes or new left bundle branch block [LBBB]);

• Development of pathological ECG Q waves;

• Imaging evidence of a new loss of viable myocardium or a new regional wall motion abnormality.

2. Coronary intervention-related MI was arbitrarily defined as an increase in cTn levels to more than five times the 99^th^ percentile of the URL in patients with normal baseline values. In patients high pre-procedure cTn levels in whom the level is stable (<20% variation) or falling, the post-procedure cTn level must rise by >20%; however, the absolute post-procedure value must still be at least five times the 99^th^ percentile of the URL.

In addition, one of the following is required:

• New ischemic ECG changes;

• The development of new pathological Q waves;

• Imaging evidence of a new loss of viable myocardium or a new regional wall motion abnormality in a pattern consistent with an ischemic etiology;

• Angiographic findings consistent with a flow-limiting procedural complication such as coronary dissection, the occlusion of a major epicardial artery or a side branch occlusion/thrombus, the disruption of collateral flow, or distal embolisation.

3. CABG-related MI was arbitrarily defined as an increase in cTn levels to >10 times the 99^th^ percentile of the URL in patients with normal baseline cTn levels. In patients with hig pre-procedure cTn levels in whom the level is stable (<20% variation) or falling, the post-procedure cTn level must rise by >20%; however, the absolute post-procedure level must still be >10 times the 99^th^ percentile of the URL. In addition, one of the following is required:

• The development of new pathological Q waves;

• Angiography documented new graft or new native coronary artery occlusion;

• Imaging evidence of a new loss of viable myocardium or a new regional wall motion abnormality in a pattern consistent with an ischemic etiology.

4. Pathological findings of an acute myocardial infarction.

**Stroke**

Stroke^2^ was defined as an acute episode of neurological dysfunction attributable to a central nervous system vascular cause. It had to be documented by computed tomography (CT) or magnetic resonance imaging (MRI) or autoptic evidence. Only ischemic stroke was considered for the primary endpoint, and was defined as an acute episode of focal brain, spinal or retinal dysfunction caused by an infarction of central nervous system tissue and documented by imaging.

**Hospitalisation for revascularisation**

Hospitalisation for revascularisation was defined as any period of hospitalisation (except for an acute myocardial infarction) during which the patient underwent percutaneous coronary revascularisation or coronary artery by-pass surgery regardless of whether or not it was prompted by clinical or functional signs of ischemia.

The clinical or functional signs of ischemia include any of the following:

a. A history of typical symptoms suggestive of stable angina pectoris;

b. Evidence of ischemia as result of a provocative test (myocardial SPECT, exercise test, stress echocardiography);

c. Abnormal results of any invasive functional diagnostic test (coronary flow reserve or fractional flow reserve).

**References**

1. [Thygesen K](https://www.ncbi.nlm.nih.gov/pubmed/?term=Thygesen%20K%5BAuthor%5D&cauthor=true&cauthor_uid=30165617), [Alpert JS](https://www.ncbi.nlm.nih.gov/pubmed/?term=Alpert%20JS%5BAuthor%5D&cauthor=true&cauthor_uid=30165617), [Jaffe AS](https://www.ncbi.nlm.nih.gov/pubmed/?term=Jaffe%20AS%5BAuthor%5D&cauthor=true&cauthor_uid=30165617), [Chaitman BR](https://www.ncbi.nlm.nih.gov/pubmed/?term=Chaitman%20BR%5BAuthor%5D&cauthor=true&cauthor_uid=30165617), [Bax JJ](https://www.ncbi.nlm.nih.gov/pubmed/?term=Bax%20JJ%5BAuthor%5D&cauthor=true&cauthor_uid=30165617), [Morrow DA](https://www.ncbi.nlm.nih.gov/pubmed/?term=Morrow%20DA%5BAuthor%5D&cauthor=true&cauthor_uid=30165617), [White HD](https://www.ncbi.nlm.nih.gov/pubmed/?term=White%20HD%5BAuthor%5D&cauthor=true&cauthor_uid=30165617); [ESC Scientific Document Group](https://www.ncbi.nlm.nih.gov/pubmed/?term=ESC%20Scientific%20Document%20Group%5BCorporate%20Author%5D). Fourth universal definition of myocardial infarction (2018). [Eur Heart J.](https://www.ncbi.nlm.nih.gov/pubmed/30165617) 2018 Aug 25. doi: 10.1093/eurheartj/ehy462.
2. Adams HP Jr, del Zoppo G, Alberts MJ et al. Guidelines for the early management of adults with ischemic stroke. Stroke 2007; 38 (5): 1655-1711.
